# Supplementary material for: Deep learning for the detection of benign and malignant pulmonary nodules in non-screening chest CT scans
Source: Commun Med (Lond). 2023 Oct 27;3:156. doi: 10.1038/s43856-023-00388-5 (PMC10611755; doi:10.1038/s43856-023-00388-5)
Supplement: Supplementary file 1 — Supplementary Information [file 43856_2023_388_MOESM1_ESM.pdf]

Supplementary information

Title

Deep learning for the detection of benign and malignant pulmonary nodules in non-screening chest CT scans

Authors

Ward Hendrix ● Nils Hendrix ● Ernst T. Scholten ● Mariëlle Mourits ● Joline Trap-de Jong ● Steven Schalekamp ● Mike Korst ● Maarten van Leuken ● Bram van Ginneken ● Mathias Prokop ● Matthieu Rutten ● Colin Jacobs

Table of contents

Supplementary Note 1 - Development and evaluation of the nodule detection system on LUNA16 ... 2

Supplementary Note 2 - Description of the nodule detection pipeline 4

Supplementary Note 3 - Annotation protocols for training data 8

Supplementary Note 4 - Analysis of the subcomponents of the nodule detection system 9

Supplementary Note 5 – Evaluation of the nodule detection performance in contrast-enhanced and non-contrast enhanced CT scans 10

References 11

Tables 13

Figures 23

Supplementary Note 1 - Development and evaluation of the nodule detection system on LUNA16

As preliminary research, we first developed and validated a pulmonary nodule detection system on the publicly available LUNA16 dataset[1]. This dataset was extended with nodule segmentations from the LIDC-IDRI archive[2]. Each CT scan in the dataset was assessed by four thoracic radiologists[2]. The details of the system architecture and training procedure are given in Supplementary Note 2. For training the nodule detection component, the pre-processed dataset was used from Fedorov et al.[3], who converted the file formats of the nodule segmentations of the LIDC-IDRI archive from XML to DICOM-SEG. All segmentations from at least one radiologist were used for training (n=2,281), the union of the segmentations was taken when multiple radiologists segmented the same nodule. Bounding boxes were derived from the nodule segmentations.

The complete pulmonary nodule detection system was evaluated with the nodule labels (diameter ≥ 3 mm) annotated by at least three radiologists (n=1,186). The nodule center coordinates and diameters are provided in the LUNA16 dataset, together with an exclusion list with abnormal, but irrelevant findings. Detections on these findings are considered as neither false positives nor false negatives. The system was evaluated with a 10-fold cross validation: we trained and tested ten models on ten different splits of the dataset, 80% of the data in each split was for training, 10% for evaluation and hyperparameter tuning, and 10% for testing. The procedure for the Free Receiver Operating Characteristic (FROC) analysis is described in the main text (see Methods).

The results of the FROC analysis are presented in Supplementary Table 1 and are visualized in Supplementary Figure 1, which also shows the performance of the subcomponents (candidate detection and false positive reduction networks). For the false positive thresholds of 0.125, 0.25, 0.5, 1, 2, 4, and 8, the complete system obtained a sensitivity of 86.0%, 90.1%, 94.4%, 96.1%, 97.7%, 98.6%, and 98.7%, respectively. The average sensitivity at all false positive rates (CPM score) was 94.5%. The maximum sensitivity of 98.8% is reached at a false positive threshold of 17.4. These

results are in line with previous pulmonary nodule detection studies, which have reported sensitivities the range of 88.0%-93.6% at 1 FP/s and CPM scores of 77.5%-92.5%[4].

Supplementary Note 2 - Description of the nodule detection pipeline

As described in the main text, the nodule detection pipeline consists of three components for the following tasks: (1) lung detection, (2) nodule candidate detection, (3) and false positive reduction. In the upcoming sections, each component will be discussed in detail. The entire pipeline was implemented with the PyTorch machine learning framework (1.9.1, 2021)[5] on a NVIDIA GeForce GTX 1080 Ti graphics card.

Lung detection model

The lung detection model is a 2D object detector with the YOLOv5 architecture (version 5.0, 2021)[6]. The main reasons for choosing an object detection model over a segmentation model was to reduce our annotation efforts and to enable faster processing speeds, albeit at the cost of localization precision[7]. An alternative approach could be to use a thresholding-based segmentation model that is commonly used in nodule detection pipelines[8–10], which requires no labelled samples. However, this method only performs well in case of absent or minimal presence of lung pathologies[11]. In a routine clinical setting, pathological conditions are usually present (e.g., consolidations, pleural effusions, fibrosis) and therefore these traditional methods can be fragile.

For preprocessing, all slices were first extracted from the CT scan. The voxel spacing in the X and Y directions were linearly resampled to an isotropic resolution of 1 mm. The intensity values of the slices were normalized by using a window size of 1500 Hounsfield Units (HU) and level of -400 HU. The image was zero-padded or a center crop was taken to meet the desired resolution of 512 x 512 pixels. The gray-scale images were converted to 8-bit RGB images.

We selected the lightweight YOLOv5s model that is pretrained on the COCO dataset[12]. The model size (i.e., small, medium, large, or extra-large) was determined empirically: for each task, we started with the lightest model configuration and increased the model capacity until no performance gain could be achieved on the validation set. The lung detection training dataset (see main body) was

used for developing the model: 80% of the CT scans was used for training (47,507 samples in total, 31,948 with bounding boxes) and 20% for validation (12,374 samples in total, 7,900 with bounding boxes). The model was trained with bounding boxes for two classes, namely the left and right lung. Translation (max ± 10%) and scaling (max ± 50%) augmentations were applied during training. An SGD optimizer with Nesterov momentum was used to update the network during training (initial learning = 1e-3, momentum = 0.937, and weight decay = 5e-4). The model was trained until convergence, that is, when the default fitness score did no longer improve on the validation set.

At inference time, the intersection over union (IoU) threshold for non-maximum suppression (NMS) was set to 0.6 and the output probability threshold to 0.6.

Nodule candidate detection model

The pulmonary nodule candidate detector is also a 2D object detector with the YOLOv5 architecture (version 5.0, 2021)[6]. Although nodule segmentation labels were available, we decided to choose an object detection approach in line with previous pulmonary nodule detection studies[4]. Unlike the lung detection model, the nodule candidate detector uses multichannel images with five consecutive CT slices instead of a single slice, since additional spatial information is needed to discriminate nodules from pulmonary vessels and other linear structures. Bounding boxes are predicted for the center slice.

For preprocessing, a 3D patch was taken around the lungs with a 2 mm margin, using the 2D bounding predictions from the lung detector (see previous section). The voxel spacing in the Z direction was normalized to 1 mm. The voxel spacing in the X and Y direction was variable: the 3D lung patch was upsampled and padded (with value of -1000 HU that corresponds to air) to obtain a resolution of 1024 x 1024 pixels. Considering that object detectors struggle with detecting small objects[13], this method maximizes the sizes of the pulmonary nodules. The same intensity normalization procedure was applied as the one for the lung detector.

We selected the large YOLOv5l model that is pretrained on the COCO dataset[12]. In comparison with the lung detection model, more model capacity was needed to fully utilize the multi-slice input images. The nodule detection training dataset was used for model development (see main body). Bounding boxes were derived from the nodule segmentations. Undersampling was applied to obtain a balanced dataset: 80% of the CT scans was used for training (48,007 samples in total, 23,791 with bounding boxes) and 20% was used for validation (12,409 samples, from which 6,430 with bounding boxes). The training procedure was largely the same as the one for the lung detector, only with a few exceptions: we also applied horizontal flipping augmentations (50% probability), subsampling of slice thickness (50% probability, max. 3 mm) and voxel spacing in the Z direction (50% probability, max. 3 mm), and increased the initial learning to 1e-3.

At inference time, samples are generated by shifting a window of 5 mm in the Z direction of the scan with a stride of 1 mm and padding of 2 mm (value = -1000 HU). For each sample, the model predicts 2D bounding boxes (IoU threshold for NMS was set to 0.1), which are clustered together by the DBSCAN algorithm (with $\varepsilon = 3$)[14]. A custom 3D non-maximum suppression (NMS) algorithm was applied to reduce the number of duplicate detections. This algorithm works similarly to the conventional NMS algorithm, but the IoU threshold is replaced by our nodule detection criterion (see main body). The final nodule center world coordinates are obtained by taking the weighted average of the predicted bounding boxes (based on the output probabilities). Nodule candidates whose center coordinate lies outside any 2D lung bounding box are discarded before the false positive reduction procedure.

Nodule false positive reduction model

The nodule false positive reduction (FPR) model is adapted from the work of Venkadesh et al.[15], which is a multi-view Resnet50 model that processes nine different 2D views from a 3D nodule patch. The only difference is that we did not incorporate the i3D classification network[16] and averaged the output probabilities of the FPR model with those from the nodule candidate detector

instead (see previous section). We selected all nodule candidates with a minimum probability threshold of 1e-2, which gave the highest sensitivity with an average false positive rate below 30.

For preprocessing, a 76x76x76 mm patch was taken from the original CT scan. Padding with value -1000 HU was applied if necessary. After applying data augmentations (see next paragraph), a 50x50x50 mm center crop was taken from the patch and resampled to obtain an isotropic voxel spacing of 0.78 mm (patch size is 64x64x64 pixels). The voxel values were normalized in the same way as the lung and nodule detection components.

We used 80% of the CT scans for training (41,349 patches in total, 3,756 with nodules) and 20% for validation (10,003 patches in total, 1,028 with nodules). During training, a stratified data sampler was used to mitigate the large class imbalance. Rotations (from -20 to +20 degrees around each axis) and horizontal and vertical translations (max. 1 mm in all directions) were randomly applied during training. In addition, the slice thickness and spacing in the Z direction were randomly subsampled (50% probability, max. 3 mm). We used an SGD optimizer with Nesterov momentum, the learning rate and momentum was set to 1e-4 and 0.9 respectively. A scheduler was used that reduced the learning rate by a factor 10 when the training loss did not decrease for 10 epochs. The model was trained until convergence, that is, when the area under the receiver operating characteristic (ROC) curve did no longer improve on the validation set.

At inference time, test time augmentations were applied with -10 or +10 degrees rotations around each axis (creating eight different combinations). The average nodule output probability was taken from the different augmentations and averaged again with the nodule output probability from the nodule candidate detector.

Supplementary Note 3 - Annotation protocols for training data

Lung bounding box annotation

We extracted every 10th slice from the CT scans from the lung detection training dataset (see main body). The pixel values were normalized with a window size of 1500 HU and level of -400 HU. A medical student annotated all slices by drawing a single bounding box per lung that encloses all lung parenchymal tissue. The maximum tolerable error was two millimetres from the lung parenchyma. In case of consolidations or pleural effusions, only the unaffected lung parenchymal tissue was selected. We used the software Visual Geometry Group (VGG) Image Annotator (version 2.0.11, 2021)[17] for annotating the bounding boxes.

Pulmonary nodule detection and segmentation

Three medical students segmented all pulmonary nodules with a diameter ≥ 3 and ≤ 30 mm in the CT scans from the training dataset (see main body). In addition, they labelled the type of the nodule (solid, part-solid, non-solid, perifissural, or calcified) and the lobe in which the nodule was located. Airway nodules were not annotated in this study. All annotations were made with in-house software (version 19.9.2 of CIRRUS Lung Screening, DIAG, Radboudumc, Nijmegen, The Netherlands).

Regarding the annotation procedure, the training data was equally divided among the three annotators. They were first asked to locate the nodules mentioned in the radiology report and then to look for remaining nodules in the scan. After the first annotation round, the pulmonary nodule detection system (trained on the LIDC-IDRI subset of the training dataset, see Supplementary Table 3) was employed to find any missed nodules. One of the annotators checked the false positive detections above a probability threshold that approximately corresponded to an average false positive rate of 4 on the LUNA16 dataset (see Supplementary Note 1). A final check of the annotations was done by the author W.H. under supervision of the radiologist M.R., who also assessed the image quality criteria as described in the main text.

Supplementary Note 4 - Analysis of the subcomponents of the nodule detection system

Evaluation of the lung detection system

We developed and evaluated a slice-by-slice lung detection system based on the YOLOv5 architecture[6]. A description of the model architecture and training procedure are provided in Supplementary Note 2. The location of the lungs was annotated in the internal and external test datasets according to the procedure as described in Supplementary Note 3. An overview of the label distribution is provided in Supplementary Table 7. The lung detection performance was evaluated by measuring the average precision and sensitivity, which were calculated per CT scan and then averaged over all scans. Predicted lung bounding boxes were counted as true positives if they had a minimum IoU of 0.9, otherwise it was considered as a false negative. At inference time, the model output probability threshold was to 0.6 and the IoU threshold for NMS was set to 0.6.

In Supplementary Table 8, the precision and sensitivity scores are reported for the left lung, right lung, and both lungs on the test datasets. The standard deviation values represent the deviation across the CT scans. The precision and sensitivity for the detection of the lungs for hospital A was 96.9 ± 2.3% and 96.8 ± 2.5%, for hospital B this was 96.8 ± 3.4% and 96.6 ± 3.8%. For hospitals A and B combined, the detection systems obtained a precision of 96.9 ± 2.9% and a sensitivity of 96.7 ± 3.2%.

Evaluation of the nodule candidate detection and false positive reduction components

We evaluated the performance of the nodule candidate detection and false positive reduction components on the test datasets (data characteristics are included in Table 1 and 2 in the main body). These results are summarized in Supplementary Table 9.

Supplementary Note 5 – Evaluation of the nodule detection performance in contrast-enhanced and non-contrast enhanced CT scans

The pulmonary nodule detection system was trained and evaluated on a mixed cohort with both contrast-enhanced and non-contrast CT scans (see Table 1 in the main text). The internal (n=100) and external test set (n=100) contained respectively 68 and 67 contrast-enhanced CT scans. Pulmonary nodules adjacent to vascular structures can be better identified and delineated in contrast-enhanced CT scans compared to non-contrast CT scans[18]. Therefore, a subgroup analysis was conducted to compare the nodule detection performance between contrast-enhanced and non-contrast CT scans. The detection performance was evaluated with a FROC analysis as described in the main text (see Methods). We evaluated the detection performance for different nodule sizes with a minimum diameter of 3, 4, and 5 mm.

The results of the FROC analysis are presented in Supplementary Table 10. These results show that the model performs slightly better for non-contrast CT scans compared to contrast-enhanced CT scans. For the internal test set, the sensitivity for detecting all nodules in contrast-enhanced CT scans at an average of 1 FP/s was 89.6% (240/268, 95% CI: 86.6%-92.5%) and the CPM was 84.5%. For non-enhanced CT scans, the sensitivity for detecting all nodules at an average of 1 FP/s was 96.1% (49/51, 95% CI: 90.3%-100.0%) and the CPM was 88.6%. For the external test set, the sensitivity for detecting all nodules in contrast-enhanced CT scans at an average of 1 FP/s was 91.9% (217/236, 95% CI: 88.9%-94.8%) and the CPM was 87.4%. For non-enhanced CT scans, the sensitivity for detecting all nodules at an average of 1 FP/s was 94.0% (63/67, 95% CI: 87.2%-98.7%) and the CPM was 88.1%. The differences between the subgroups were similar for the different nodule sizes. It is important to note that contrast-enhanced CT scans are ordered for estimating the malignancy of nodules[19]. Hence, malignant nodules were not evenly distributed over the subgroups (e.g., all patients with pulmonary metastases received intravenous contrast), and that is a confounding factor that influenced the subgroup analysis.

References

1. Setio, A. A. A. *et al.* Validation, comparison, and combination of algorithms for automatic detection of pulmonary nodules in computed tomography images: the LUNA16 challenge. *Medical Image Analysis* **42**, 1–13 (2016).
2. Armato, S. G. *et al.* The Lung Image Database Consortium (LIDC) and Image Database Resource Initiative (IDRI): A completed reference database of lung nodules on CT scans. *Medical Physics* **38**, 915–931 (2011).
3. Fedorov, A. *et al.* DICOM re-encoding of volumetrically annotated Lung Imaging Database Consortium (LIDC) nodules. *Medical Physics* **47**, 5953–5965 (2020).
4. Gu, Y. *et al.* A survey of computer-aided diagnosis of lung nodules from CT scans using deep learning. *Computers in Biology and Medicine* **137**, 104806 (2021).
5. Paszke, A. *et al.* PyTorch: An Imperative Style, High-Performance Deep Learning Library. Preprint at https://doi.org/10.48550/arXiv.1912.01703 (2019).
6. Jocher, G. *et al.* ultralytics/yolov5: v5.0 - YOLOv5-P6 1280 models, AWS, Supervise.ly and YouTube integrations. (2021) doi:10.5281/zenodo.4679653.
7. Wang, Y., Ahsan, U., Li, H. & Hagen, M. A Comprehensive Review of Modern Object Segmentation Approaches. *FNT in Computer Graphics and Vision* **13**, 111–283 (2022).
8. Liao, F., Liang, M., Li, Z., Hu, X. & Song, S. Evaluate the Malignancy of Pulmonary Nodules Using the 3-D Deep Leaky Noisy-OR Network. *IEEE Transactions on Neural Networks and Learning Systems* **30**, 3484–3495 (2019).
9. Han, Y. *et al.* Pulmonary nodules detection assistant platform: An effective computer aided system for early pulmonary nodules detection in physical examination. *Computer Methods and Programs in Biomedicine* **217**, 106680 (2022).
10. Zheng, S. *et al.* Automatic Pulmonary Nodule Detection in CT Scans Using Convolutional Neural Networks Based on Maximum Intensity Projection. *IEEE Transactions on Medical Imaging* **39**, 797–805 (2020).

11. Mansoor, A. *et al.* Segmentation and Image Analysis of Abnormal Lungs at CT: Current Approaches, Challenges, and Future Trends. *RadioGraphics* **35**, 1056–1076 (2015).
12. Lin, T.-Y. *et al.* Microsoft COCO: Common Objects in Context. Preprint at https://doi.org/10.48550/arXiv.1405.0312 (2015).
13. Benjumea, A., Teeti, I., Cuzzolin, F. & Bradley, A. YOLO-Z: Improving small object detection in YOLOv5 for autonomous vehicles. Preprint at https://doi.org/10.48550/arXiv.2112.11798 (2023).
14. Ester, M., Kriegel, H.-P., Sander, J. & Xu, X. A density-based algorithm for discovering clusters in large spatial databases with noise. in *Proceedings of the Second International Conference on Knowledge Discovery and Data Mining* 226–231 (AAAI Press, 1996).
15. Venkadesh, K. V. *et al.* Deep Learning for Malignancy Risk Estimation of Pulmonary Nodules Detected at Low-Dose Screening CT. *Radiology* **300**, 438–447 (2021).
16. Carreira, J. & Zisserman, A. Quo Vadis, Action Recognition? A New Model and the Kinetics Dataset. Preprint at https://doi.org/10.48550/arXiv.1705.07750 (2018).
17. Dutta, A. & Zisserman, A. The VIA Annotation Software for Images, Audio and Video. in *Proceedings of the 27th ACM International Conference on Multimedia* 2276–2279 (Association for Computing Machinery, 2019). doi:10.1145/3343031.3350535.
18. Purysko, C. P., Renapurkar, R. & Bolen, M. A. When does chest CT require contrast enhancement? *CCJM* **83**, 423–426 (2016).
19. Ohno, Y. *et al.* Dynamic Contrast-Enhanced CT and MRI for Pulmonary Nodule Assessment. *American Journal of Roentgenology* **202**, 515–529 (2014).
20. Brierley, J. D., Gospodarowicz, M. K. & Wittekind, C. *TNM classification of malignant tumours*. (John Wiley & Sons, 2016).
21. Hendrix, W. *et al.* Trends in the incidence of pulmonary nodules in chest computed tomography: 10-year results from two Dutch hospitals. *Eur Radiol* (2023) doi:10.1007/s00330-023-09826-3.

Tables

Supplementary Table 1. Pulmonary nodule detection results on the LUNA16 dataset (1,186 nodules, 888 scans), reported per system component and complete system. The model sensitivity (%) is reported for each false positive rate.

	**Average number of false positives per scan**							
	**0.125**	**0.25**	**0.5**	**1**	**2**	**4**	**8**	**CPM**
Candidate detection model	54.2	71.9	86.7	92.5	95.8	97.3	97.9	85.2
False positive reduction model	75.5	85.7	90.1	93.8	96.3	97.9	98.3	91.1
Complete system	86.0	90.1	94.4	96.1	97.7	98.6	98.7	94.5

Abbreviations: CPM = Competition performance metric, the average sensitivity at all false positive rates.

Supplementary Table 2. Additional imaging parameters for the training and testing datasets.

Dataset	**Lung detection training dataset**	**Nodule detection training dataset**	**Internal test set**	**External test set**
Source(s)	Hospital A + LIDC/IDRI	Hospital A + LIDC/IDRI	Hospital A	Hospital B
CT scans	1,388	1,490	100	100
Peak potential energy in kVp (mean, range)	118 (80-140)	117 (100-140)	109 (100-135)	108 (100-120)
Tube current in mAs (mean, range)	223 (10-688)	208 (10-650)	220 (10-649)	287 (23-787)
Scanners (n, % of total scans)				
Canon				
Aquilion One	266 (19.2)	285 (19.1)	44 (44.0)	
Aquilion Precision	120 (8.6)	195 (13.1)	26 (26.0)	
Aquilion CXL	107 (7.7)	127 (8.5)	17 (17.0)	
GE Healthcare				
LightSpeed 16	197 (14.2)	197 (13.2)		
LightSpeed Ultra	162 (11.7)	162 (10.9)		
LightSpeed QX/i	97 (7.0)	97 (6.5)		
LightSpeed pro 16	79 (5.7)	79 (5.3)		
LightSpeed VCT	61 (4.4)	61 (4.1)		
LightSpeed Plus	56 (4.0)	56 (3.8)		
Philips				
Brilliance 16P	54 (3.9)	54 (3.6)		
Brilliance iCT 256			3 (3.0)	
Siemens				
Somatom Definition Flash				44 (44.0)
Somatom Definition AS+				29 (29.0)
Somatom Edge				6 (6.0)
Sensation 16	95 (6.8)	95 (6.4)		
Sensation 64	49 (3.5)	49 (3.3)		19 (19.0)
Biograph 40			6 (6.0)	
Other	45 (3.2)	33 (2.2)	4 (4.0)	2 (2.0)

Note. For the training datasets, we selected the same subset of CT scans as used in the LUNA16 challenge[1]. The Other category contains less than 5% of all CT scanners in the specified dataset.

Supplementary Table 3. Characteristics of the pulmonary nodules in the training dataset, reported per subset.

Subset	**LIDC-IDRI[1]**	**Hospital A**
CT scans	888	602
Total nodules[2]	2,281	2,489
Nodules per diameter threshold (n, % of total)[3,4]		
≥ 4 mm	1,896 (83.1)	1,680 (67.5)
≥ 5 mm	1,326 (58.1)	1,127 (45.3)
Diameter (in mm)[3]		
Median	5.4	4.7
IQR	4.3-7.6	3.7-6.5
Volume (in mm^3)[3]		
Median	82.8	55.0
IQR	43.1-235.3	26.4-147.2
Nodules per scan		
Median	2	3
IQR	1-3	1-6
Nodules per type (n, % of total)[5]		
Solid	1,398 (61.3)	1,341 (53.9)
Part-solid	354 (15.5)	95 (3.8)
Non-solid	309 (13.5)	160 (6.4)
Perifissural	N/A	684 (27.5)
Calcified	220 (9.6)	209 (8.4)

[1] We selected the same subset of CT scans as used in the LUNA16 challenge[1]. [2] Pulmonary nodules are labelled by at least one annotator. [3] In case of multiple annotations, the volume and equivalent diameter labels from the different readers were averaged per nodule. Interquartile range (IQR) is from the 25th to the 75th percentile. [4] There are 37 nodules in the LIDC-IDRI dataset with a diameter < 3 mm when using the equivalent diameters instead of the manually measured diameters. [5] For the LIDC-IDRI dataset, a value of 2 out of 5 was considered as non-solid and a value of 4 out of 5 as part-solid. Perifissural nodules were not labelled in the LIDC-IDRI dataset.

Supplementary Table 4. Characteristics of the malignant nodules in the internal (hospital A) and external (hospital B) test set.

Dataset	**Internal test dataset**		**External test dataset**	
Primary lung cancer or metastasis	Primary lung cancer	Pulmonary metastasis	Primary lung cancer	Pulmonary metastasis
Total nodules	27	165	32	113
Total patients	27	25	28	25
Equivalent diameter (in mm)				
Median	17.2	5.1	18.3	7.3
IQR	13.7-21.6	3.9-7.0	13.8-22.3	4.9-12.7
Volume (in mm^3) per percentile				
Median	2,703	71	3,245	221
IQR	1,403-5,468	31-180	1,383-6,026	61-1,184
Nodules per scan				
Median	1	4	1	3
IQR	1-1	1-9	1-1	1-7
Nodule location (n, % of total nodules)				
Left lower lobe	4 (14.8)	47 (28.5)	3 (9.4)	30 (26.5)
Left upper lobe	4 (14.8)	27 (16.4)	5 (15.6)	36 (31.9)
Right lower lobe	5 (18.5)	58 (35.2)	4 (12.5)	21 (18.6)
Right middle lobe		11 (6.7)	2 (6.2)	8 (7.1)
Right upper lobe	14 (51.9)	22 (13.3)	18 (56.2)	18 (15.9)

Note. The volume and equivalent diameter labels from the different readers were averaged per nodule. Interquartile range (IQR) is from the 25th to the 75th percentile.

Supplementary Table 5. Cancer diagnosis characteristics from the patients in the internal (hospital A) and external (hospital B) test set.

Dataset	**Internal test dataset**		**External test dataset**	
Cancer type	Lung cancer	Extrapulmonary cancer	Lung cancer	Extrapulmonary cancer
Total patients	36	14	35	15
Total diagnoses	36	14	37	15
Involves pulmonary metastases (n, % of total diagnoses)	11 (30.6)	14 (100)	10 (27.0)	15 (100)
Stage (n, % of total diagnoses)				
IA	19 (52.8)		13 (35.1)	
IB	6 (16.7)		14 (37.8)	
II		1 (7.1)		
III		1 (7.1)		
IV	11 (30.6)	10 (71.4)	10 (27.0)	12 (80.0)
N/A[1]		2 (14.3)		3 (20.0)
Histologic type (n, % of total diagnoses)[2]				
Adenocarcinoma	23 (63.9)	11 (78.6)	23 (62.2)	10 (66.7)
Squamous-cell carcinoma	8 (22.2)	1 (7.1)	12 (32.4)	2 (13.3)
Large-cell carcinoma	2 (5.6)			
Small-cell carcinoma	1 (2.8)		1 (2.7)	
Other	2 (5.6)	2 (14.3)	1 (2.7)	3 (20.0)
Basis of diagnosis (n, % of diagnoses per site)				
Primary site				
Histological examination	25 (100)		27 (100)	
Clinical diagnostic testing[3]				
Hematology/cytology testing[4]				
Metastasis site (lung)				
Histological examination	1 (9.1)	1 (7.1)	1 (10.0)	1 (6.7)
Clinical diagnostic testing[3]	10 (90.9)	13 (92.9)	9 (90.0)	14 (93.3)
Hematology/cytology testing[4]				

[1] Tumour, Node, Metastasis (TNM) staging is not applicable for the type of tumour at the time of diagnosis. [2] The exact classification of the histologic type of the cancers can be found in Supplementary Table 6. [3] For example: medical imaging, exploratory surgery, or autopsy (without confirmation by microscopy). [4] For example: bone marrow aspiration, blood testing, or any diagnosis confirmed by microscopy, but unclear whether it concerns cytology or histology.

Supplementary Table 6. Histology categories of the cancers in the test datasets based on ICD-O morphology codes[20], separately reported for the internal (hospital A) and external (hospital B) test set.

Dataset	**Internal test set**		**External test set**	
Cancer type	Lung cancer	Extrapulmonary cancer	Lung cancer	Extrapulmonary cancer
Total diagnoses	36	14	37	15
Histologic type (n, % of total diagnoses)				
Adenocarcinoma				
8140	9 (25.0)	6 (42.9)	8 (21.6)	9 (60.0)
8145		1 (7.1)		
8170		1 (7.1)		
8250	9 (25.0)		3 (8.1)	
8252	1 (2.8)			
8257	1 (2.8)			
8260		1 (7.1)		
8310		1 (7.1)		1 (6.7)
8550	3 (8.3)		8 (21.6)	
8551			4 (10.8)	
8941		1 (7.1)		
Squamous-cell carcinoma				
8070	4 (11.1)		9 (24.3)	2 (13.3)
8071	1 (2.8)			
8072	3 (8.3)		2 (5.4)	
8074			1 (2.7)	
8086		1 (7.1)		
Large-cell carcinoma				
8046	2 (5.6)			
Small-cell carcinoma				
8041	1 (2.8)		1 (2.7)	
Other				
8000	1 (2.8)		1 (2.7)	2 (13.3)
8240	1 (2.8)			
8980				1 (6.7)
9085		1 (7.1)		
9180		1 (7.1)		

Supplementary Table 7. Characteristics of the lung bounding boxes in the internal (hospital A) and external (hospital B) test set.

	Internal test set (hospital A)	External test set (hospital B)
Total CT scans	100	100
Annotated slices	8,057	3,012
Bounding boxes (n, % of total)		
Any lung	9,953	3,728
Left lung	4,932	1,843
Right lung	5,021	1,885

Supplementary Table 8. Precision (%) and sensitivity (%) of the slice-by-slice lung detection system on the internal (hospital A) and external (hospital B) test set.

	Internal test set (hospital A)		External test set (hospital B)	
	**Precision**	**Sensitivity**	**Precision**	**Sensitivity**
Any lung	96.9 (2.3)	96.8 (2.5)	96.8 (3.4)	96.6 (3.8)
Left lung	96.9 (3.7)	96.7 (4.4)	97.8 (4.0)	97.6 (4.6)
Right lung	96.9 (2.6)	96.8 (2.6)	95.8 (5.4)	95.6 (5.6)

Note. Precision and sensitivity are calculated per CT scan and then averaged over all scans. Standard deviations are in parentheses and represent the deviation across the CT scans.

Supplementary Table 9. Pulmonary nodule detection results of each model component on the internal (hospital A) and external (hospital B) test set. The model sensitivity (%) is reported for each false positive rate for all nodules ≥ 3 mm.

	**Average number of false positives per scan**							
	**0.125**	**0.25**	**0.5**	**1**	**2**	**4**	**8**	**CPM**
Internal (hospital A)								
Candidate detection	41.7	59.6	77.6	88.4	94.2	97.7	99.2	79.8
False positive reduction	62.5	70.9	80.0	86.5	94.2	97.7	99.2	84.4
Combined	70.3	81.9	90.0	94.2	97.3	98.8	99.2	90.2
External (hospital B)								
Candidate detection	43.3	59.7	69.8	83.6	90.3	96.3	97.8	77.2
False positive reduction	60.8	76.1	81.3	87.3	95.3	96.3	98.1	85.0
Combined	71.6	80.9	88.5	94.8	95.9	97.8	98.5	89.7

Abbreviations: CPM = Competition performance metric, the average sensitivity at all false positive rates.

Supplementary Table 10. Pulmonary nodule detection results for contrast-enhanced and non-contrast CT scans on the internal (hospital A) and external (hospital B) test set. The model sensitivity (%) is reported for each false positive rate.

		**Average number of false positives per scan**							
	**Count**	**0.125**	**0.25**	**0.5**	**1**	**2**	**4**	**8**	**CPM**
Internal (hospital A)									
Contrast-enhanced									
Nodules ≥ 3 mm	268	60.4	70.0	82.1	89.6	94.4	96.6	98.5	84.5
Nodules ≥ 4 mm	207	72.9	80.4	89.9	91.8	96.1	97.1	98.6	89.5
Nodules ≥ 5 mm	154	77.9	82.1	89.6	90.9	95.5	96.8	98.7	90.2
Non-contrast									
Nodules ≥ 3 mm	51	65.0	78.4	82.6	96.1	98.0	100.0	100.0	88.6
Nodules ≥ 4 mm	43	69.6	79.1	84.0	97.7	100.0	100.0	100.0	90.1
Nodules ≥ 5 mm	34	74.1	82.4	88.6	97.1	100.0	100.0	100.0	91.7
External (hospital B)									
Contrast-enhanced									
Nodules ≥ 3 mm	236	69.9	76.3	86.0	91.9	94.5	96.6	96.6	87.4
Nodules ≥ 4 mm	203	76.8	81.8	89.2	93.6	96.1	97.0	97.0	90.2
Nodules ≥ 5 mm	166	78.9	83.1	89.8	94.0	97.0	97.6	97.6	91.1
Non-contrast									
Nodules ≥ 3 mm	67	62.7	79.1	89.6	94.0	94.0	97.0	100.0	88.1
Nodules ≥ 4 mm	59	69.5	88.1	94.9	96.6	96.6	98.3	100.0	92.0
Nodules ≥ 5 mm	49	73.5	89.8	95.9	95.9	95.9	98.0	100.0	92.7

Abbreviations: CPM = Competition performance metric, the average sensitivity at all false positive rates.

Figures

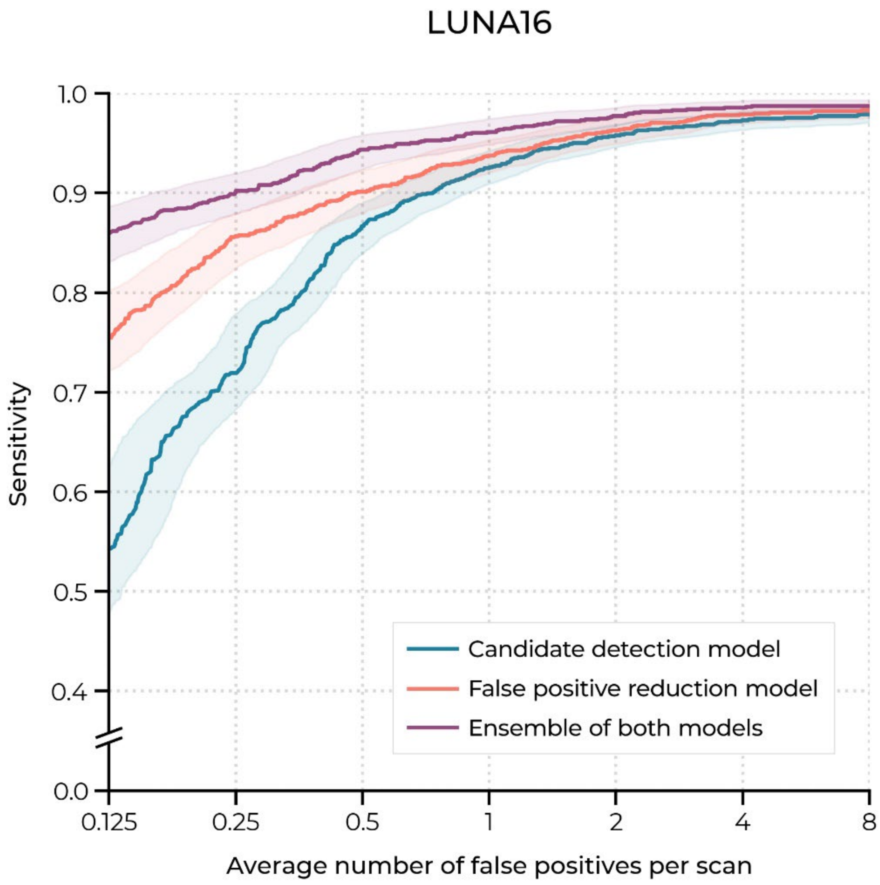

Supplementary Figure 1. Free Receiver Operating Characteristic (FROC) analysis of the overall pulmonary nodule detection system and its subcomponents on the LUNA16 dataset with 10-fold cross validation (1,186 nodules, 888 scans). The shaded bands represent the 95% confidence intervals per configuration.

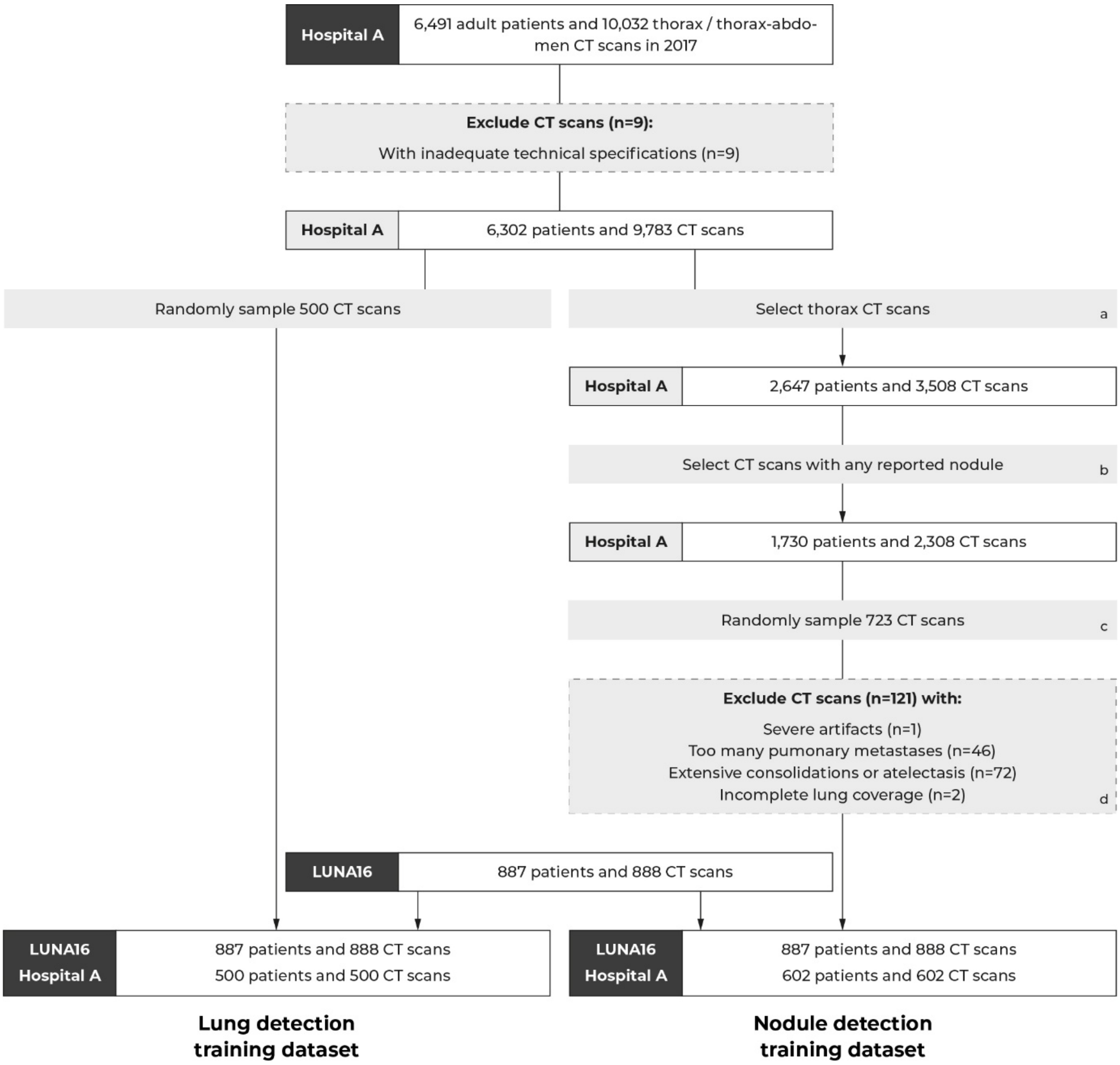

Supplementary Figure 2. Flowchart for creating the training datasets for the valuation of the lung and nodule detection components. [a] Only thorax CT scans were selected to facilitate the nodule annotation process. [b] Based on a natural language processing analysis[21]. [c] One CT scan per patient was sampled. [d] Visual inspection was done by the author W.H. under supervision of the radiologist M.R.
