# Supplementary material for: Deep learning for the detection of benign and malignant pulmonary nodules in non-screening chest CT scans
Source: Commun Med (Lond). 2023 Oct 27;3:156. doi: 10.1038/s43856-023-00388-5 (PMC10611755; doi:10.1038/s43856-023-00388-5)
Supplement: Supplementary file 4 — Description of Additional Supplementary Files [file 43856_2023_388_MOESM4_ESM.pdf]

Description of Additional Supplementary Files

File name: Supplementary Data 1
Description: Numerical results underlying the graphs in Figure 3.

File name: Supplementary Data 2
Description: Numerical results underlying the graphs in Figure 6.
