# Supplementary material for: Deep learning for the detection of benign and malignant pulmonary nodules in non-screening chest CT scans
Source: Commun Med (Lond). 2023 Oct 27;3:156. doi: 10.1038/s43856-023-00388-5 (PMC10611755; doi:10.1038/s43856-023-00388-5)
Supplement: Supplementary file 6 — Reporting Summary [file 43856_2023_388_MOESM6_ESM.pdf]

nature portfolio

Corresponding author(s): Colin Jacobs

Last updated by author(s): Sep 29, 2023

Reporting Summary

Nature Portfolio wishes to improve the reproducibility of the work that we publish. This form provides structure for consistency and transparency in reporting. For further information on Nature Portfolio policies, see our Editorial Policies and the Editorial Policy Checklist.

Statistics

For all statistical analyses, confirm that the following items are present in the figure legend, table legend, main text, or Methods section.

n/a	Confirmed	
☐	☒	The exact sample size (n) for each experimental group/condition, given as a discrete number and unit of measurement
☐	☒	A statement on whether measurements were taken from distinct samples or whether the same sample was measured repeatedly
☐	☒	The statistical test(s) used AND whether they are one- or two-sided<br>*Only common tests should be described solely by name; describe more complex techniques in the Methods section.*
☒	☐	A description of all covariates tested
☒	☐	A description of any assumptions or corrections, such as tests of normality and adjustment for multiple comparisons
☐	☒	A full description of the statistical parameters including central tendency (e.g. means) or other basic estimates (e.g. regression coefficient) AND variation (e.g. standard deviation) or associated estimates of uncertainty (e.g. confidence intervals)
☐	☒	For null hypothesis testing, the test statistic (e.g. F, t, r) with confidence intervals, effect sizes, degrees of freedom and P value noted<br>*Give P values as exact values whenever suitable.*
☒	☐	For Bayesian analysis, information on the choice of priors and Markov chain Monte Carlo settings
☒	☐	For hierarchical and complex designs, identification of the appropriate level for tests and full reporting of outcomes
☐	☒	Estimates of effect sizes (e.g. Cohen's d, Pearson's r), indicating how they were calculated

Our web collection on statistics for biologists contains articles on many of the points above.

Software and code

Policy information about availability of computer code

Data collection	Data was collected from the picture archiving and communications systems (PACS) from the hospitals and the publicly available LUNA16 challenge dataset (10.5281/zenodo.3723295 and 10.5281/zenodo.4121926). For annotating the lung bounding boxes, we used the software Visual Geometry Group (VGG) Image Annotator (version 2.0.11, DOI: 10.1145/3343031.3350535). We used in-house software (version 19.9.2 of CIRRUS Lung Screening, DIAG, Radboudumc, Nijmegen, The Netherlands) to segment pulmonary nodules in the private datasets. We used the re-encoded DICOM nodule segmentations from Federov et al. (DOI: 10.1002/mp.14445) that correspond to the CT scans from the LUNA16 challenge. The data processing details are described in the supplementary material.
Data analysis	The proposed system is freely available for research purposes at the platform Grand-Challenge (https://grand-challenge.org/algorithms/lung-nodule-detector-for-ct/). The lung and nodule candidate detection models are based on the YOLOv5 architecture (version 5.0, DOI: 10.5281/zenodo.467965331). The nodule false positive reduction model was adapted from the previous work from our group (DOI: 10.1148/radiol.202120443335). The model implementation details are described in the supplementary material. Code for the Free Receiver Operating Characteristic (FROC) analysis is available at the LUNA16 challenge website (https://luna16.grand-challenge.org/Evaluation/). Significance testing was performed with the MLxtend library for Python (version 0.22.0, DOI: 10.21105/joss.0063836).

For manuscripts utilizing custom algorithms or software that are central to the research but not yet described in published literature, software must be made available to editors and reviewers. We strongly encourage code deposition in a community repository (e.g. GitHub). See the Nature Portfolio guidelines for submitting code & software for further information.

Data

Policy information about availability of data

All manuscripts must include a data availability statement. This statement should provide the following information, where applicable:

- Accession codes, unique identifiers, or web links for publicly available datasets
- A description of any restrictions on data availability
- For clinical datasets or third party data, please ensure that the statement adheres to our policy

Data from the LUNA16 challenge is available via the Cancer Imaging Archive (DOI: 10.7937/K9/TCIA.2015.LO9QL9SX) and Zenodo (https://zenodo.org/record/2595813 and https://zenodo.org/record/4121926). Clinical data collected at the Radboud University Medical Centre and Jeroen Bosch Hospital are not released publicly, but can be requested from the investigators. Reasonable requests for de-identified data for research purposes will be considered by the corresponding author and requires approval from the institutional review boards before access. Numerical results underlying the graphs in Figures 3 and 6 are available in Supplementary Data 1 and Supplementary Data 2, respectively.

Human research participants

Policy information about studies involving human research participants and Sex and Gender in Research.

Reporting on sex and gender	For the private datasets, we collected the patient sex from the electronic health records (if available).
Population characteristics	In accordance with the British Thoracic Society (BTS) nodule management guidelines, only adult patients (≥ 18 years old) were included. For the selection primary lung cancer cases, we included patients with stage I cancer as they include nodules instead of masses (> 30 mm). For the selection of pulmonary metastases cases, both patients with metastasized lung cancer and extra-thoracic cancer were included. For the internal test set (100 patients), the average patient age was 63 ± 15 years (52 women). For the external test set (100 patients), the average patient age was 67 ± 12 years (53 women).
Recruitment	We included all patients patients who underwent a chest CT scan in the period 2017-2020 (retrospective study design).
Ethics oversight	At all institutions, the institutional review board waived the need for informed consent because of the retrospective design and the use of anonymized data in this study.

Note that full information on the approval of the study protocol must also be provided in the manuscript.

Field-specific reporting

Please select the one below that is the best fit for your research. If you are not sure, read the appropriate sections before making your selection.

☒ Life sciences ☐ Behavioural & social sciences ☐ Ecological, evolutionary & environmental sciences

For a reference copy of the document with all sections, see nature.com/documents/nr-reporting-summary-flat.pdf

Life sciences study design

All studies must disclose on these points even when the disclosure is negative.

Sample size	The sample size of the private trainings data obtained the hospital A were based on the size of the LUNA16 dataset (see Supplementary Table 3). For the test sets, we aimed to collect 100 scans per hospital, which was mainly determined by the maximum number of scans that could be annotated by the panel of radiologists.
Data exclusions	Considering the routine clinical setting of our study, it is important to note that not all patients can be reliably screened for malignant pulmonary nodules. Patients with extensive fibrosis or consolidations (e.g., due to severe interstitial diseases, hemorrhage, or pneumonia) were excluded, as their lungs contain high attenuation areas that prevent correct location and delineation of relevant nodules. Furthermore, patients were excluded if CT scans were made with a slice thickness > 3 mm, or were limited by severe breathing artifacts or incomplete coverage of the lungs. Finally, patients with more than 15 pulmonary metastases (according to the initial visual assessment) were excluded to reduce annotation efforts and prevent data imbalance.
Replication	Given the retrospective design of our study, all experimental findings can be reproduced. The AI models developed in this study are saved on Grand-Challenge (https://grand-challenge.org/algorithms/) where the model output can be validated and assessed.
Randomization	For the development of the private training datasets, CT scans with and without reported pulmonary nodules were randomly sampled. For the development of the test datasets, CT scans were evenly, randomly sampled from four categories to obtain a balanced datasets: (1) patients with stage I lung cancer; (2) patients with pulmonary metastases; (3) patients with benign pulmonary nodules larger than 5 mm for which imaging follow-up would be recommended; and (4) patients with benign pulmonary nodules smaller than 5 mm or no nodules (which were considered as “normal”).
Blinding	Blinding is not applicable to our study design.

Reporting for specific materials, systems and methods

We require information from authors about some types of materials, experimental systems and methods used in many studies. Here, indicate whether each material, system or method listed is relevant to your study. If you are not sure if a list item applies to your research, read the appropriate section before selecting a response.

Materials & experimental systems

n/a	Involved in the study
☒	☐ Antibodies
☒	☐ Eukaryotic cell lines
☒	☐ Palaeontology and archaeology
☒	☐ Animals and other organisms
☒	☐ Clinical data
☒	☐ Dual use research of concern

Methods

n/a	Involved in the study
☒	☐ ChIP-seq
☒	☐ Flow cytometry
☒	☐ MRI-based neuroimaging
